# Mortality Rates Among Individuals Diagnosed with Pemphigus: 12-Year Experience in Tehran, Iran

**DOI:** 10.34172/aim.31996

**Published:** 2025-02-01

**Authors:** Nastaran Namazi, Fahimeh Abdollahimajd, Ghazal Mardani, Zahra Razzaghi, Hamideh Moravvej

**Affiliations:** ^1^Skin Research Center, Shahid Beheshti University of Medical Sciences, Tehran, Iran; ^2^Laser Application in Medical Sciences Research Center, Shahid Beheshti University of Medical Sciences, Tehran, Iran

**Keywords:** Comorbidities, Corticosteroids, Mortality, Pemphigus, Rituximab

## Abstract

**Background:**

Pemphigus consists of a group of rare autoimmune bullous diseases that affect the skin and mucous membranes. Pemphigus includes three major forms: pemphigus vulgaris (PV), pemphigus foliaceus, and paraneoplastic pemphigus. Before the advent of systemic corticosteroids (SCSs), pemphigus was usually a fatal disease. Rituximab (RTX), a monoclonal antibody against the CD20+B cells has been approved for the treatment of patients with pemphigus. Previous studies have confirmed the high efficacy and safety profile of RTX in pemphigus patients. We aimed to estimate the overall mortality and causes of death among pemphigus patients who were admitted to the hospitals of Shahid Beheshti University of medical Sciences, Tehran, Iran, before and after administering RTX.

**Methods:**

We included 480 patients admitted to Shahid Beheshti University of medical Sciences hospitals, Tehran, Iran, from October 2010 to October 2022. The diagnosis of all patients was confirmed by direct immunofluorescence and pathological studies. All variables such as age, sex, type of pemphigus, presence of comorbidities, medications, and cause of death were assessed.

**Results:**

The prevalence of pemphigus was 262 (54.58%) in women and 218 (45.41%) in men (*P* value=0.004). These included 474 (98.75%) PV, 4 (0.83%) pemphigus foliaceus and 2 (0.41%) paraneoplastic pemphigus cases. The most common comorbidities were hypertension and diabetes mellitus (98 [20.41%] and 93 [19.37%], respectively). The overall mortality was 20 (4.16%), including 15 (75%) patients under treatment with high dose SCSs and immunosuppressive agents, and 5 (25%) patients who received at least 500 mg of RTX and low dose SCSs.

**Conclusion:**

The mean age of the disease was found to be a decade earlier than other parts of the world, with a higher preponderance of women. The most common comorbidities were hypertension and diabetes mellitus. Most deaths were due to infectious and cardiovascular diseases. Mortality rate was 1/3 in patients who received RTX compared to those who were treated with high dose corticosteroids and other immunosuppressive agents.

## Introduction

 Pemphigus encompasses a group of rare chronic autoimmune blistering diseases of the skin and mucous membranes. Pemphigus includes three major forms: pemphigus vulgaris (PV), pemphigus foliaceus and paraneoplastic pemphigus. PV and pemphigus foliaceus account for 75% and 20% of cases, respectively.^1^ The desmosomal cadherins desmoglein 1 and 3 have been identified as autoantigens in PV and desmoglein 1 in pemphigus foliaceus.^2^

 PV is a potentially fatal disease that appears as flaccid blisters on the mucosae and skin that lead to painful erosions.^3^ PV lesions are characterized by intraepidermal vesicles with acantholysis and intact basal layer, with IgG autoantibodies directed against desmosomal cadherins desmoglein-1 and desmoglein-3 antigens, which indicate a role as adhesive factors between nearby keratinocytes.^4,5^ The incidence of PV varies in different parts of the world, and Iran is recognized as a high-incidence country for PV.^3,6-8^ PV is the most common type of pemphigus that accounts for 80% of all cases^8^ and exhibits variability in clinical presentations, epidemiologic characteristics, and severity.^3^

 Systemic corticosteroids (SCSs) and immunosuppressive therapy are traditional treatments for pemphigus.^9^ Although the prognosis of PV has been improved, pemphigus stills remain a life-threatening autoimmune disease.^9,10^ In addition, long-term use of SCSs and immunosuppressants may be associated with different morbidities.^11^ Rituximab (RTX), a monoclonal antibody against CD20 + B cells, has been approved for treatment of patients with PV.^12^ Previous studies have confirmed the high efficacy and safety of RTX in pemphigus.^13,14^

 This study is designed to evaluate the mortality and morbidity rate and cause-specific mortality of pemphigus patients before and after administration of RTX in dermatology centers of Shahid Beheshti University of Medical Sciences, Tehran, Iran, between 2010 and 2022.

## Materials and Methods

 We conducted a 12-year retrospective study on clinical patterns of 480 patients with pemphigus referred to the dermatology departments of Shohadaye Tajrish and Loghman Hakim Hospital affiliated to Shahid Beheshti University of Medical Sciences, Tehran, Iran, from January 2010 to December 2022.

 Diagnosis was confirmed by histopathological and immunopathological criteria (detection of IgG or IgA intercellular deposits at direct immunofluorescence microscopy from a perilesional tissue sample). The medical data including demographic data, past medical history, drug history, clinical variant of pemphigus, duration of disease, presence of comorbidities and mortality reports were extracted from the Shahid Beheshti University of Medical Sciences registry.

 Data were analyzed with SPSS software (IBM SPSS Statistics for Windows, Version 26.0. Armonk, NY: IBM Corp). Quantitative variables were described with mean ± SD, normality evaluation was done with Shapiro-Wilks test, and two groups of variables were compared using independent sample t-test. Qualitative variables were described with frequency (percent), and chi-square or Fisher’s exact test was used for comparing data. A *P* value less than 0.05 was considered significant.

## Results

 Four-hundred eighty patients were evaluated in this study, of whom 218 (45.41%) were male and 262 (54.58%) were female (*P* value = 0.049). The mean age of patients at disease onset was 43.71 ± 14.13 years and it was the same for males and females (*P* value = 0.294).

 Patients in the age group of 40-49 years showed the highest risk of pemphigus development (74 [15.4%] and 59 [10.8%] for women and men, respectively), while patients in the age group under 20 years had the lowest incidence of pemphigus development (1 [0.2%] and 3 [0.6%] for women and men, respectively). The distribution of patients by age group and gender is shown in Table 1.

**Table 1:** Distribution of Patients by Age Group and Gender

**Age Group (y)**	**Female**		**Male**	
< 20	N = 1	0.2%	N = 3	0.6%
20-29	N = 16	3.3%	N = 21	4.4%
30-39	N = 57	11.9%	N = 49	10.2%
40-49	N = 74	15. 4%	N = 52	10.8%
50-59	N = 67	14%	N = 52	10.8%
≥ 60	N = 47	9.8%	N = 41	8.5%
Total	N = 262	54.6%	N = 218	45.3%

 The average disease duration was 3.83 (0.1-46) years. The mean duration of hospitalization was 7.71 (1-53) days. We found 474 (98.75%) PV, 4 (0.83%) pemphigus foliaceus and 2 (0.41%) paraneoplastic pemphigus cases. At the time of diagnosis, 330 (68.75%) patients had mucosal lesions; in the remaining cases, the disease presented initially on the skin. In the most of the cases, prednisone and/or azathioprine, cyclosporine, mycophenolate mofetil, and cyclophosphamide had been administered before the administration of RTX. Comorbidities are outlined in Table 2.

**Table 2:** Comorbidities in Patients with Pemphigus

**Comorbidities**	**Number**	**Percent**
Hypertension	98	20.4
Diabetes mellitus	93	19.3
Hyperlipidemia	80	16.6
Cardiovascular disease	26	5.4
Hypothyroidism	25	5.2
Mood disorder	24	5
Anxiety disorder	20	4.1
Chronic kidney disease	7	1.4
Obstructive pulmonary disease	5	1.04
Peptic ulcer disease	3	0.6
Cerebrovascular disease	3	0.6
Rheumatoid arthritis	3	0.6
Epilepsy	1	0.2
Thyrotoxicosis	1	0.2
Myasthenia gravis	1	0.2
Avascular necrosis of femur	1	0.2
Multiple sclerosis	1	0.2
Chronic lymphocytic leukemia	1	0.2
Parkinsonism	1	0.2
Autoimmune hepatitis	1	0.2

 Twenty (4.16%) patients died during the study; among them, 15 (75%) were under treatment with high-dose SCSs ( > 40-mg daily) and immunosuppressive agents, while 5 (25%) had received at least 500 mg of RTX + /- low dose ( < 40-mg daily) SCS. The number of deaths was 13 (65%) in males and 7 (35%) in females (*P* value = 0.072). Also, 7 (35%) of the deceased patients and 206 (44.8%) of the living patients had comorbidities (*P* value = 0.389).

 The patients’ demographic and clinical characteristics, treatment and causes of death are provided in Table 3.

**Table 3:** Patients’ Demographics, Duration, Type of Pemphigus, Comorbidities, Treatment and Causes of Death

**Age (y)/Sex**	**Duration**	**Comorbidities**	**Type of Pemphigus**	**Treatment Given**	**Antecedent Causes of Death**
90/M	1 mon	Non	PV	High dose SCS	Pulmonary TB reactivation
82/F	2 mon	HTN, DM	PV	High dose SCS, azathioprine	Pancreatitis
63/M	3 y	Non	PV	High dose SCS, mycophenolate mofetil	Cardiac arrest
50/F	6 y	HTN, MDD	PV	RTX, low dose SCS	Necrotizing fasciitis
75/M	9 mon	Non	PV	RTX, IVIG, high dose SCS	Sepsis
58/F	18 mon	HTN	PV	High dose SCS, azathioprine, IVIG	PTE
81/M	3 mon	Hypothyroidism, IHD, HTN	PV	High dose SCS	Ruptured Aortic aneurysm
52/F	3 y	HTN, DM	PV	High dose SCS, mycophenolate, mofetil, IVIG, plasmapheresis, dapsone, RTX, cyclophosphamide	COVID-19 pneumonia
51/M	3 mon	Non	PV	High dose SCS, azathioprine	Sepsis
37/M	1 y	MDD	PV	High dose SCS, azathioprine	Suicide
38/F	3 y	Hypothyroidism, HLP	PV	High dose SCS, azathioprine	Sepsis
51/M	1 mon	Non	PV	High dose SCS, azathioprine	GI bleeding
57/M	3 mon	HTN, DM, HLP	PV	High dose SCS, azathioprine	GI bleeding
60/M	3 mon	CLL, DM, HTN	PP	SCS, RTX	Sepsis
65/M	2 y	HTN, DM,	PV	High dose SCS, azathioprine, IVIG	Sepsis
67/M	4 mon	Non	PV	SCS, cyclophosphamide, azathioprine, RTX	Sepsis
67/M	6 mon	MDD	PV	High dose SCS, cyclophosphamide	PTE
68/F	1 mon	HTN, DM, HLP	PV	High dose SCS, azathioprine	Sepsis
71/M	3 y	DM, CKD, IHD	PV	High dose SCS, dapsone, azathioprine	Cardiac arrest
85/F	1 mon	HTN	PV	High dose SCS, azathioprine, IVIG	Sepsis

M: Male, F: Female, PV: Pemphigus vulgaris, SCS: Systemic corticosteroid, RTX: Rituximab, IVIG: Intravenous immunoglobulin, TB: Tuberculosis, PTE: Pulmonary thromboembolism, GI: Gastrointestinal.

Table 4 demonstrates the causes of death in patients with pemphigus during the 12 years of the study. Most deaths were attributed to infectious diseases, including pneumonia and septicemia. Other causes were GI bleeding and cardiovascular disease.

**Table 4:** Causes of Death in Patients with Pemphigus

**Cause of Death**	**Number**	**Percent **
Pneumonia and sepsis	8	40
Cardiovascular disease	2	10
Gastrointestinal bleeding	2	10
Pulmonary thromboembolism	2	10
Ruptured aortic aneurysm	1	5
COVID-19 pneumonia	1	5
Necrotizing fasciitis	1	5
Pancreatitis	1	5
Suicide	1	5
Pulmonary tuberculosis reactivation	1	5

## Discussion

 PV is the most common form of pemphigus with an uneven geographic distribution.^8,15-19^ The current study indicated a relatively higher incidence of pemphigus in women than men, consistent with the reports from Iranian patients in other medical centers of Iran.^3,7,8,20,21^ The mean age of onset of pemphigus in our patients was 47.58 ± 13.22 and 46.31 ± 13.19 years for women and men, respectively, which corresponds to data from other parts of Iran and Taiwan.^3,7-9^ In our study, the preponderance of PV was observed among the age group 40–49 years, and the lowest incidence of pemphigus was observed in patients who were in the age group of under 20 years.

 The mortality rate in our study was 4.1%, which is different from a study performed on 55 pemphigus patients in Kerman, a southern province of Iran, which reported a total of 11 deaths (20%). Chams-Davatchi et al reported a 6.2% death rate in 1209 pemphigus patients, in Razi hospital, Tehran, Iran, during 19 years of patient follow-up.^3,7^

 In a cohort study by Huang et al in Taiwan on 853 pemphigus patients during 2002-2009, 88 deaths (10%) were observed. They reported that most deaths were due to pneumonia, septicemia, cardiovascular disease, and peptic ulcer which is consistent with our study. Their study revealed that patients with pemphigus had twice higher mortality rates compared to the general population, with a much higher rate of mortality among older patients aged above 60 years.^9^ In our study, most mortalities were in cases aged above 50 years.

 In a 19-year retrospective evaluation of pemphigus in a single dermatology center in Istanbul, Turkey, between December 1995 and December 2014, nine deaths were reported in 196 patients. The mortality rate was 6%.^22^

 In a cohort study by Kridin et al on 245 patients with pemphigus in Haifa, Israel, 48 deaths (19%) were reported during a mean follow-up period of 10.9 ± 8.1 years, with infectious diseases, namely pneumonia and septicemia, and cardiovascular diseases as the most common causes, which is consistent with our results. Their findings showed that overall mortality among pemphigus patients is 2.4 times higher than the general population.^23^

 A study in France reported 66 deaths among 249 pemphigus patients over a 10-year period (26%), which was mainly correlated with the old age of cases.^24^

 Our study indicated that survival in patients with pemphigus was higher in women compared to men. Our study showed that PV was the predominant subtype of pemphigus (98.93% of patients) compared to foliaceus pemphigus (0.83%) and paraneoplastic pemphigus (0.41%) in two referral hospitals in Tehran, Iran.

 Numerous studies have indicated the safety and clinical consequences of RTX in the treatment of refractory autoimmune disease.^25-27^ Although in our study, five patients died after RTX treatment (three with sepsis, one with necrotizing fasciitis, one with COVID-19 pneumonia), the mortality rate of PV was much lower in patients who received RTX (25%) compared to those who were treated with SCSs and immunosuppressive agents, suggesting that RTX can be used as a lifesaving treatment in pemphigus patients.

## Conclusion

 In our study, PV was the most common type of pemphigus. The incidence of PV was higher among women than men. Moreover, patients belonging to the age group of 40-49 showed the highest risk of developing PV in the two-referral hospitals of Shahid Beheshti University of Medical Sciences, Tehran, Iran (Shohadaye Tajrish and Loghman Hakim hospitals). Most deaths were due to infectious diseases (pneumonia and septicemia) and cardiovascular diseases. The death rate of PV was much lower in patients who received RTX compared to those who were treated with SCSs and immunosuppressive agents.
